# Natural Hybridization and Introgression between *Ligularia cymbulifera* and *L. tongolensis* (Asteraceae, Senecioneae) in Four Different Locations

**DOI:** 10.1371/journal.pone.0115167

**Published:** 2014-12-31

**Authors:** Jiaojun Yu, Chiaki Kuroda, Xun Gong

**Affiliations:** 1 Key Laboratory for Plant Diversity and Biogeography of East Asia, Kunming Institute of Botany, Chinese Academy of Sciences, Kunming, P. R. China; 2 University of Chinese Academy of Sciences, Beijing, P. R. China; 3 Department of Chemistry, Rikkyo University, Tokyo, Japan; Institute of Botany, China

## Abstract

Natural hybridization has been considered to represent an important factor influencing the high diversity of the genus *Ligularia* Cass. in the Hengduan Mountains, China. Natural hybridization has been confirmed to occur frequently in *Ligularia*. To date, however, it has been demonstrated only within a single population. In this paper, we present evidence of natural hybridization in *Ligularia* from four different locations. The internal transcribed spacer (ITS) region of the nuclear ribosomal DNA and three chloroplast intergenic spacers (*trn*K-*rps*16, *trn*L-*rpl*32 and *trn*Q-5'*rps*16) of 149 accessions of putative hybrids and their putative parents (*L. cymbulifera* and *L. tongolensis*) were analyzed for evidence of hybridization. The ITS data clearly distinguished two putative parental species and sympatric *L. vellerea* and supported the hypothesis that those morphological intermediates were products of natural hybridization between *L. cymbulifera* and *L. tongolensis*. Moreover, several identified morphological parents were actual introgressed products. Because of hybridization and introgression, chloroplast DNA sequences generated a poorly resolved network. The present results indicate that varying degrees of hybridization and introgression occur differently depending on the habitat context. We conclude that gene flow caused by natural hybridization in *Ligularia* indeed plays an important role in the species diversity.

## Introduction

The importance of hybridization in plant speciation and evolution has been debated for decades, with opposing views of hybridization as either a creative evolutionary force or evolutionary noise [1]–[8]. Natural hybridization may occur at either the homoploid (i.e., between two species of the same ploidy) or the polyploid level, each with its attendant genetic and evolutionary consequences. The consequences of hybridization may include introgression affecting one or both taxa, the formation of hybrid (especially alloploid) species, or the development of reticulate patterns of evolution within a group [1], [5].

The genus *Ligularia* Cass. [9], [10] consists of approximately 120 species mainly distributed in eastern Asia, 112 species of which are known in China [10]. The majority of *Ligularia* distributed in the Hengduan Mountains region are endemic [9], [11]. The Qinghai-Tibetan Plateau (QTP), adjacent and closely related to the Hengduan Mountains region because of the collision between India and Eurasia, is one of the important alpine biodiversity hotspots. The mechanisms for plant diversification on the QTP include, e.g., allopatric speciation via geographic isolation, diploid hybridization and introgression, and allopolyploidy [12], [13]. The high species diversity of the genus *Ligularia* is considered to have arisen from rapid and continuous allopatric speciation in small and isolated populations, combined with interspecific diploid hybridization in the QTP and adjacent areas of eastern Asia [11], [14]. Natural hybrids have commonly been found in certain areas populated by *Ligularia* [11], [15], [16]. *Ligularia* × *maoniushanensis* X. Gong & Y. Z. Pan is the first natural hybrid reported as a result of comprehensive study [17]. Furthermore, based on an examination of the chemical similarities of *L. lamarum* (Diels) C. C. Chang and *L. subspicata* (Bureau & Franchet) Handel-Mazzetti, Saito et al. [18] presumed that the two species hybridize with other *Ligularia* species in certain populations. A study on natural hybridization between *L. subspicata* and *L. vellerea* (Franchet) Handel-Mazzetti confirmed this conjecture [16]. Yu et al. [15] studied a mixed location with 6 *Ligularia* species. Based on morphological and molecular data (cpDNA and ITS sequences, ISSR markers), bidirectional but asymmetric hybridization and introgression were detected between *L. subspicata* and *L. nelumbifolia* (Bureau & Franchet) Handel-Mazzetti. Hybridization is very common in *Ligularia*, with various forms and progenies. However, these instances were all found in a single plot, and there has been no natural hybridization reported from multiple sites to date.

*Ligularia cymbulifera* (W. W. Smith) Handel-Mazzetti and *L. tongolensis* (Franchet) Handel-Mazzetti belong to the series *Lapathifolia* S. W. Liu [19]. They are widely distributed in the Hengduan Mountains, are especially abundant near Zhongdian, Yunnan, and occupy a great variety of habitats ranging from 2000 to 4000 m in altitude. Morphological intermediates are often present where the two species are sympatrically distributed. In certain populations, a portion of the individuals are different from other, typical individuals of the species. These morphological intermediates and variant individuals are considered to be progenies of crosses between *L. cymbulifera* and *L. tongolensis* based on their intermediate morphological traits and the partial overlap of the flowering period.

In the current study, the ITS region of the nuclear ribosomal DNA of all the sampled individuals was sequenced to determine the hybrid status of the morphological taxon. If hybridization was confirmed, we sequenced three chloroplast intergenic spacers (*trn*K-*rps*16, *trn*L-*rpl*32 and *trn*Q-5'*rps*16) for all collections to determine the relationship between these progenies and the two putative parents. In particular, we paid attention to the extent, direction and dynamics of introgression between the two species. In addition, we also took advantage of the existence of four independent hybrid zones to compare the outcome of hybridization in each zone and test the hypothesis that hybrid zones can evolve differently and / or result from independent hybridization processes.

## Materials and Methods

### Material collection and molecular methods

Four mixed-growing populations of *Ligularia cymbulifera*, *L. tongolensis* and their putative progenies were chosen for our study. Two other populations with only one species each (*L. cymbulifera* or *L. tongolensis*) were also chosen as references (Table S1 in S1 File, Figure S1 in S2 File). At the six locations, no specific permissions were required for scientific research. No endangered or protected species were involved at the sampling locations. Other *Ligularia* species found in these locations were collected to verify whether the genetic material of the putative hybrids had been contaminated by other materials. Plants were identified according to the diagnostic morphological characteristics according to *Flora of China* [10]. Briefly, sympatric *L. vellerea* differs from the putative parents and hybrids in terms of the raceme inflorescence and a densely long white lanate circle at the stem base. *L. cymbulifera* and *L. tongolensis* mainly differ in the size and pubescence of the leaves, the capitula number of the inflorescence and the length of the peduncle (Table 1). Young leaves were collected and dried in silica gel in the field for DNA extraction. Total genomic DNA was extracted from the silica gel–dried leaf tissue using the cetyltrimethyl ammonium bromide method [20] with minor modifications.

**Table 1:** Key morphological differences among *Ligularia cymbulifera*, *L. tongolensis* and putative hybrids.

Taxon	Leaf size	Leaf and petiole indumentum	Capitula number in inflorescence	Peduncle length
*Ligularia cymbulifera*	15–60*to 45 cm	white arachnoid-pubescent	more than 50,numerous	2–15 mm
*L. tongolensis*	3–17*2.5–12 cm	shortly pilose	less than 30	2–7 cm
putative hybrids	intermediate	white arachnoid-pubescent and shortly pilose	intermediate	5–20 mm

The ITS region of the nuclear ribosomal DNA of all the sampled individuals was amplified using primers ITS4 and ITS5 [21]. PCR was conducted in a total reaction volume of 20 µL containing 10–30 ng template DNA, 2.0 µL 10× PCR buffer with (NH_4_)_2_SO_4_, 1.3 µL MgCl_2_ (25 mM), 0.5 µL dNTP (2.5 mM each), 0.3 mM each primer and 0.75 unit of *Taq* polymerase (Takara). The amplifications were performed as follows: 1 cycle, 94°C, 5 min; 30 cycles, 94°C, 45 sec; 55°C, 1 min; 72°C, 40 sec, followed by a final extension of 1 cycle, 72°C, 5 min. The PCR products were purified by electrophoresis through a 1.2% agarose gel, and then an E.Z.N.A. Gel Extraction Kit (Omega, Guangzhou, China) was used. All accessions were subjected to sequencing with amplification primers in an ABI 3700 DNA automated sequencer with the BigDye Terminator Cycle Sequencing Kit (Applied Biosystems, Foster City, California, U.S.A.). The sequences were aligned and compared in SeqMan (DNASTAR, Beijing, China) [22]. Although direct sequencing was successful for most *L. cymbulifera* and *L. tongolensis*, it produced chimeric or unreadable peaks in the chromatograms for the putative hybrids. Hence, cloning sequencing was performed subsequently for the putative hybrid. Purified PCR products were cloned into plasmids using the pUM-T vector system (Bioteke Corporation, Beijing, China). Four to 20 positive clones were selected for each amplification product and cultured to isolate plasmids. Positive clones with the correct size inserts were confirmed using colony PCR. The plasmids with correct inserts were sequenced using ITS4 and ITS5 primers.

Four cpDNA fragments were amplified using the following universal primers: *trn*L-*rpL*32, *trn*Q-5′*rps*16, and *trn*K-*rps*16 [23]. PCR was conducted in a reaction volume of 20 µL containing 10–30 ng template DNA, 2.0 µL 10× PCR buffer with (NH_4_)_2_SO_4_, 1.0 µL MgCl_2_ (25 mM), 1.0 µL dNTP (2.5 mM each), 0.3 mM each primer and 1.5 unit of *Taq* polymerase (Takara). The amplification conditions were as follows: 1 cycle, 94°C, 4 min; 30 cycles, 94°C, 45 sec; 53°C, 45 sec; 72°C, 1 min or 50 sec, followed by 1 cycle, 72°C, 7 min. PCR products were purified and then directly sequenced using the methods mentioned above. New DNA sequences were deposited in Genbank under accession numbers KM036217-KM036281.

### Phylogenetic and network analysis

Minor variation (usually one or two nucleotide sites) between some clones may have resulted from PCR errors caused by Taq DNA polymerase. Putative PCR-mediated recombinants were excluded before further phylogenetic analysis. All sequences were aligned in Clustal X [24] complemented with PAUP 4.0. Information about variable sites was obtained using the program DnaSP 4.0 [25]. The aligned sequences were used to infer the phylogeny of the sampled individuals using the criterion of maximum parsimony, implemented in PAUP* version 4.0b [26]. Parsimony analyses were performed using a heuristic search with tree bisection and reconnection (TBR) branch swapping, the Multrees option, ACCTRAN optimization, and 1000 random addition replicates for the nuclear and chloroplast datasets. Sequences of three *Ligularia* species, *L. lingiana*, *L. subspicata*, and *L. nelumbifolia*, were downloaded from GenBank (accession numbers: JF767227-JF767229, JF767236, JF767237, JF767242, JF767272, JF767273, JF767276, JF767247, JF767248, JF767250) as outgroups in the parsimony analyses. The nrITS networks and chloroplast networks were constructed using the program TCS version 1.21 [27]. The parsimony probability was set at 95%; therefore, haplotypes related with a probability of parsimony of >95% would be connected, and those with a probability of <95% would be unlinked. The graph generated from TCS 1.21 was drawn with Adobe Illustrator (Adobe Systems, Mountain View, CA, U.S.A.).

## Results

### nrDNA ITS4-5 analysis

The length of all the ITS sequences was 753 bp, with 49 variable sites. A total of 15 haplotypes were identified (Table S2 in S1 File). *Ligularia vellerea*, which appeared in the hybrid population, showing 18 specific variable sites (at position 96, 100, 174, 202, 208, 250, 263, 275, 295, 443, 447, 506, 542, 579, 585, 606, 630, 673), could be clearly distinguished from the putative parental species and hybrids (Hap 15) (Table S2 in S1 File).

All individuals in the two reference populations (Jiajinshan and Suochonghe) were homologous and independent, as no double peaks were present in the direct sequences. For one of the putative parental species, *L. cymbulifera*, ten collections from the reference population shared haplotype H1 and H3. Except for three collections from Desha, most *L. cymbulifera* (Pc, Xc and Lc) in the mixed-growing populations were pure and shared haplotype H1, which was also present in the reference population (Table 2). These three special *L. cymbulifera* were morphologically different from typical *L. cymbulifera* in the forms of their leaf base and inflorescence. Their direct sequences were similar to those of the putative hybrids and had double peaks at more than 10 sites. The cloned sequences showed that each of the three individuals shared two haplotypes, H4 and H8. Another putative parent, *L. tongolensis*, appeared more variable than *L. cymbulifera*. Except for the mixed population from Desha, several of the *L. tongolensis* collections in the other three populations were not as pure as expected. These conflicting individuals were cloned as well. For those pure individuals (no double peaks in direct sequences), *L. tongolensis* shared haplotype H8 in Desha, H9 in Xiaoxueshan, H10 in Pachahai, and H12 in the Jiajinshan reference population and the Jiawa mixed population (Table 2). All the putative natural hybrids exhibited more than 10 double peaks, and the cloned sequences (including those inconsistent individuals of putative pure parents) mainly included two sequence types; other haplotypes not included in the pure sequences may have resulted from PCR and plasmid amplification error. The putative hybrids shared the same copies with the putative parents *L. cymbulifera* and *L. tongolensis*.

**Table 2:** Distribution of ITS4-5 haplotypes and three cpDNA haplotypes in 6 locations.

Locality	Taxa	Individuals of direct sequenced	Nuclear Haplotypes (no. of individuals)	Individuals of cloned sequenced for ITS	cpDNA Haplotypes (no. of individuals)
1. Jiajinshan, Baoxing (Sichuan)	*Ligularia tongolensis*	10	H12(10)		H6(3), H7(6), H8(1)
2. Suochonghe, Daocheng (Sichuan)	*L. cymbulifera*	10	H1(9), H3(1)		H1(3), H2(7)
3. Desha, Daocheng (Sichuan)	*L. tongolensis*	10	H8(10)		H4(1), H5(9)
	*L. cymbulifera*	3*	H4(3), H8(3)	3/3	H2(2), H3(1)
	putative hybrids	10	H1(4), H7(6), H8(10)	10/10	H2(1), H4(9)
4. Pachahai, Zhongdian (Yunnan)	*L. tongolensis*	10	H1(3), H10(7), H11(3)	2/10	H3(1), H7(3), H13(6)
	*L. cymbulifera*	10	H1(10)		H3(3), H7(6), H12(1)
	putative hybrids	10	H1(9), H2(1), H11(9), H14(1)	10/10	H7(6), H12(4)
	*L. vellerea*	10	H15(10)		H23(1), H24(5), H25(3), H26(1)
5. Xiaoxueshan, Zhongdian (Yunnan)	*L. tongolensis*	10	H1(1), H9(9), H11(1)	1/10	H21(9), H22(1)
	*L. cymbulifera*	10	H1(10)		H7(3), H14(4), H15(1), H16(1), H17(1)
	putative hybrids	12	H1(12), H10(1), H11(11)	12/12	H14(1), H15(1), H18(8), Xh19(1), Xh20(1)
	*L. vellerea*	4*	H15(4)		H25(3), H27(1)
6. Jiawa, Litang (Sichuan)	*L. tongolensis*	10	H4(2), H12(7), H13(2)	2/10	H3(1), H4(8), H7(1)
	*L. cymbulifera*	10	H1(10)		H4(9), H6(1)
	putative hybrids	10	H1(2), H4(2), H5(5), H6(1), H12(9), H13(1)	10/10	H4(5), H9(3), H10(1), H11(1)

Note:* indicates only three or four individuals for this taxon were found within the population.

**Figure 1:**
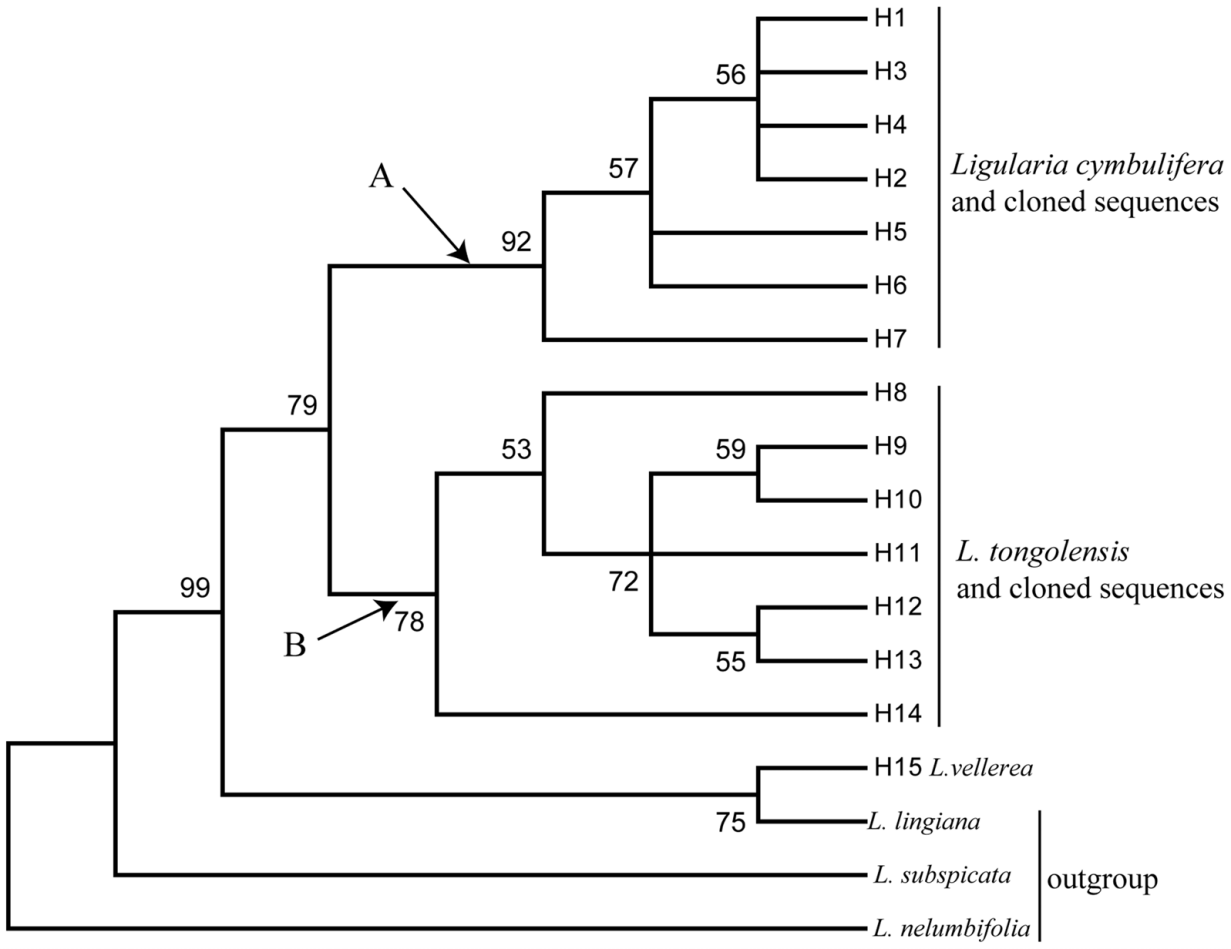
Phylogenetic relationships of the nrITS4-5 haplotypes of *L. cymbulifera*, *L. tongolensis*, their putative hybrids and sympatric *L. vellerea* from south-western China (H1–H15) generated by PAUP.

A strict consensus tree from the maximum parsimony (MP) analysis was generated to show the phylogenetic relationships (Fig. 1). The MP tree indicated that the haplotypes of putative parental species and putative hybrids formed two clusters (cluster A and B), respectively, and that sympatric *L. vellerea* formed a cluster with one of the outgroups, *L. lingiana*. In cluster A, H1 and H3 represent homologous individuals of *L. cymbulifera* that were detected in direct sequences in most locations (except for in Desha). Haplotypes H1–H7 were all detected in cloned sequences. In cluster B, H8–H10 and H12 represented homologous individuals of *L. tongolensis* that were detected in direct sequences. Haplotypes H8–H14 were all detected in cloned sequences. Among the cloned sequences (Table 2), each cloned individual possessed one haplotype that clustered with the sequences from each parental species haplotype (haplotypes). Hence, all the putative hybrid accessions are products of hybridization between *L. cymbulifera* and *L. tongolensis* [28]. Thus, sympatric *L. vellerea* is apparently not involved in the hybridization between *L. cymbulifera* and *L. tongolensis*.

**Figure 2:**
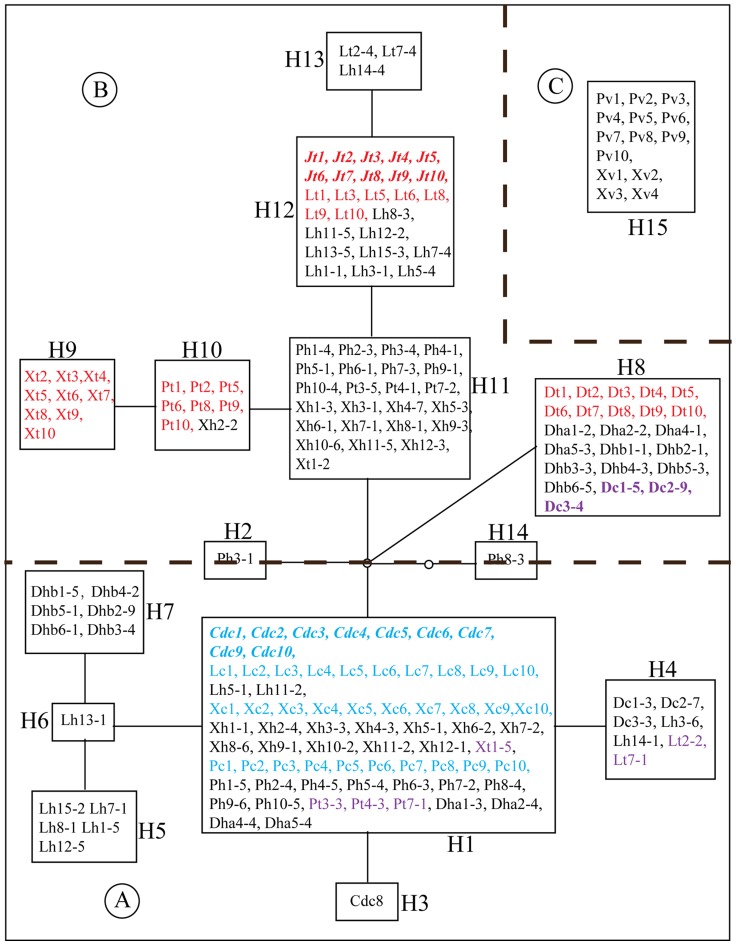
TCS networks based on nuclear ribosomal internal transcribed spacer (nrITS) sequences. Rectangular areas in the nrITS network represent nrITS haplotypes and circle areas represent haplotypes not detected. Blue and red characters represent sequences of pure individuals in reference and sympatric populations. Purple characters represent sequences of individuals possess introgressed sequences. (a) Population locality, **J**, Jiajinshan; **Cd**, Reference Daocheng; **D**, Desha; **P**, Pachahai; **X**, Xiaoxueshan; **L**, Jiawa; (b) taxa: **v**, *L. vellerea*; **t**, *L. tongolensis*; c, *L. cymbulifera*; h, putative hybrids. Numbers following taxon initials are sample numbers and clone numbers (if any). (TIFF)

The networks based on haplotypes identified from the nuclear ribosomal internal transcribed spacer (nrITS) of *L. cymbulifera*, *L. tongolensis*, their putative hybrids and sympatric *L. vellerea* were generated to show their relationships (Fig. 2). Sympatric *L. vellerea* was a completely separate component of the ITS network. Except for the totally distinct part of *L. vellerea* (part C), the nrITS network resolved two major parts (part A and B, which correspond to cluster A and B in the phylogenetic tree of Fig. 1) that were correlated with the two putative parents (*L. cymbulifera* and *L. tongolensis*) (Fig. 2). Because one ITS haplotype (H1) was shared in all the *L. cymbulifera* and most of the putative hybrids, it was the most common haplotype in the network. Part A of the network included not only the putative parental *L. cymbulifera* and hybrid clones but also several individuals of another putative parent, *L. tongolensis*. The results indicate that these individuals (X12 in Xiaoxueshan; Pt3, Pt4 and Pt7 in Pachahai; and Lt2 and Lt7 in Jiawa) of *L. tongolensis* were introgressed by *L. cymbulifera*. In part B of the network, the four mixed populations had a special haplotype. There were also three individuals (Dc1, Dc2 and Dc3) of *L. cymbulifera* having *L. tongolensis* clones, which indicates that *L. cymbulifera* were introgressed by *L. tongolensis* in Desha. In the Desha population, the number of *L. tongolensis* was much greater than that of *L. cymbulifera*, with nearly no typical *L. cymbulifera* to be found; in other populations, however, *L. cymbulifera* was very easy to recognize. Unlike the Desha population, in which individuals of *L. tongolensis* shared only one ITS haplotype (H8), *L. tongolensis* in other populations were heterogeneous. In the three mixed populations, individuals of *L. tongolensis* had different haplotypes for pure individuals; additionally, a portion of the actual introgressed individuals shared haplotypes with both *L. tongolensis* and *L. cymbulifera*. For putative hybrids, both the *L. tongolensis* and *L. cymbulifera* ITS types were found in cloned sequences.

### Combined three cpDNA intergenic spacer regions analysis

The *trn*K-*rps*16, *trn*L-*rpl*32 and *trn*Q*-*5'*rp*s16 spacers aligned sequences were 923, 928 and 978 bp, respectively. The combined length varied from 3523 to 3571 bp, containing a total of 20 variable sites (Table S3 in S1 File). Twenty-seven haplotypes (H1–H27) were identified among all collections (Table S3 in S1 File). *Ligularia vellerea*, which was sympatric with the hybrid populations (Xiaoxueshan and Pachahai) and already excluded from the possibility of participation in nuclear donors based on ITS, shared their special haplotypes H23-H27. Because chloroplast DNA is maternally inherited in *Ligularia* [29], the possibility of *L. vellerea* being a plastid donor was rejected. In contrast to the ITS4-5, each taxon in each population had more than two chloroplast haplotypes (Table 2).

**Figure 3:**
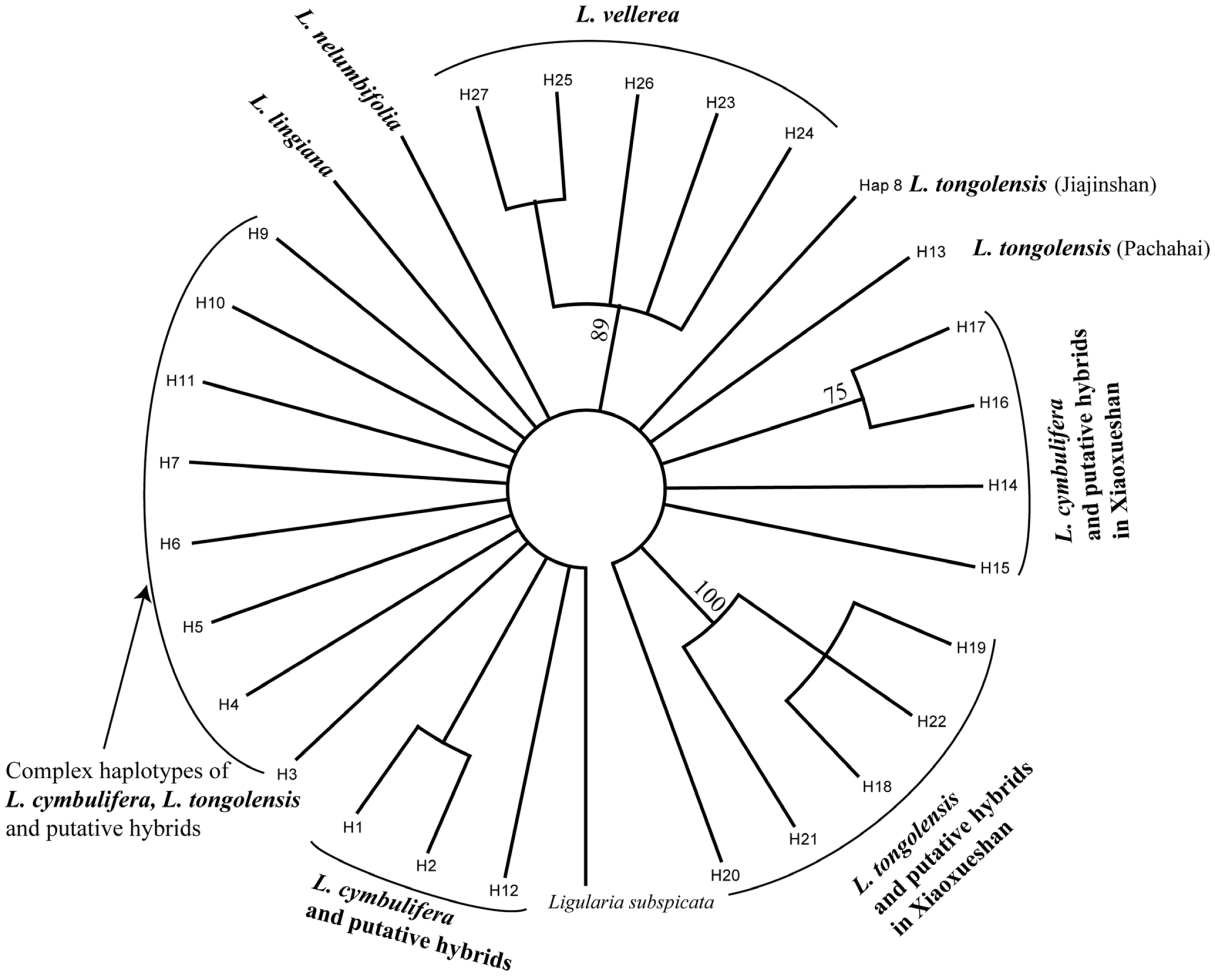
Phylogenetic relationships of the cpDNA haplotypes of *L. cymbulifera*, *L. tongolensis*, their putative hybrids and sympatric *L. vellerea* from south-western China (H1–H27) generated with PAUP.

The MP tree obtained from the four concatenated cpDNA regions is shown in Fig. 3. The tree indicates a poor phylogenetic relationship that differs from our expectations not only for sympatric *L. vellerea* but also for outgroups embedded in the haplotypes of the putative parental species and hybrids.

**Figure 4:**
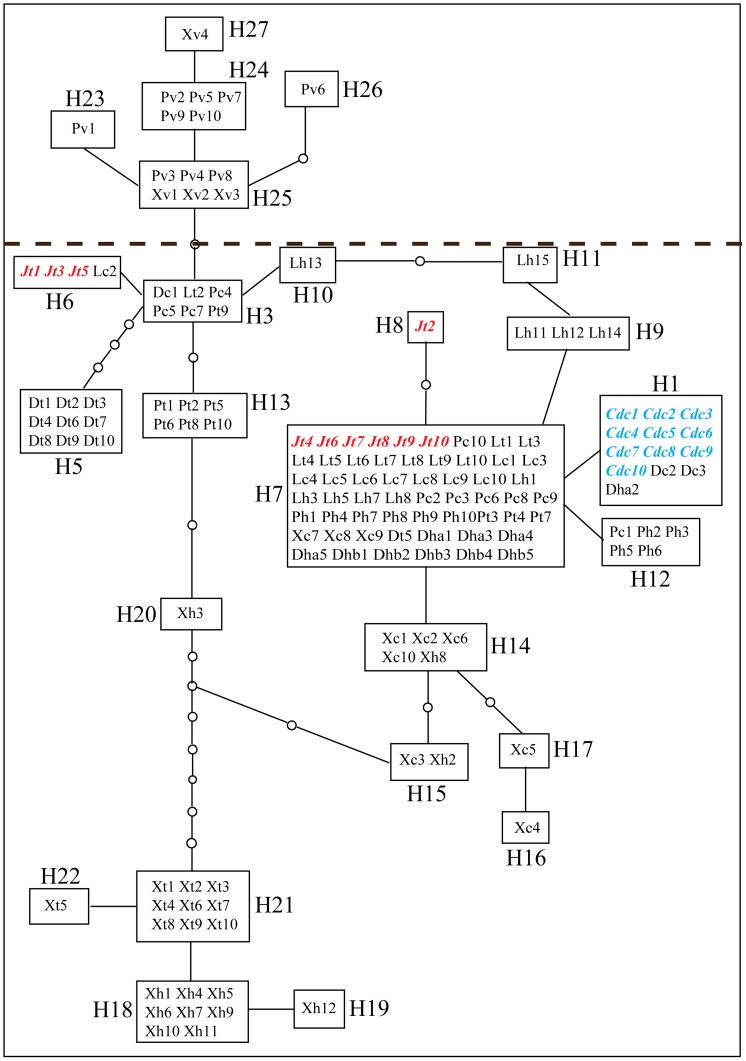
TCS haplotype networks based on plastid non-coding regions. Rectangular areas represent plastid haplotypes, and circle areas represent haplotypes not detected. Blue and red characters represent sequences of individuals in reference populations. (a) Population locality, **J**, Jiajinshan; **cd**, Reference Daocheng; **D**, Desha; **P**, Pachahai; **X**, Xiaoxueshan; **L**, Jiawa; (b) taxa: **v**, *L. vellerea*; **t**, *L. tongolensis*; c, *L. cymbulifera*; h, putative hybrids. Numbers following taxon initials are sample numbers.

The networks based on haplotypes identified from three cpDNA intergenic spacer sequences of *L. cymbulifera*, *L. tongolensis*, their putative hybrids and sympatric *L. vellerea* were generated to show their relationships (Fig. 4). Sympatric *L. vellerea* occupied a separate portion, lacking common haplotypes with the other parts of the cpDNA network. The cpDNA network appeared more confused than the ITS network. Except for the part occupied by *L. vellerea*, a large loop covered most individuals of the putative parents and hybrids (Fig. 4). The most common haplotype (H7) existed in all the four mixed populations and in almost every taxon related to the hybrids. Because hybridization and introgression were ascertained based on ITS data, the poorly resolved cpDNA network was highly plausible.

## Discussion

### Probability of occurrence of natural hybrids

Gottlieb [30] proposed several features by which natural hybridization could be identified. These features included a geographical distribution in the region of parental sympatry, morphological intermediacy in several characteristics, partial fertility, biochemical additivity, and experimental synthesis of individuals resembling the hybrid. Although no single criterion can provide a clear answer in testing a hypothesis of hybridization, each criterion that is met provides a higher level of support for a hybrid origin. In fact, the morphological characters of hybrids are not always intermediate because of the effects, e.g., of dominance, epistasis, additivity, and linkage [31]. Nevertheless, intermediate characters remain the most important clue to natural hybridization in a field study.

*L. tongolensis* and *L. cymbulifera* are both widely distributed in southwestern and western Sichuan Province, northwestern Yunnan Province and southern Xizang Province [10]. They are abundant near Zhongdian, Yunnan, and occupy a great variety of habitats ranging from 2000 to 4000 m in altitude. *L. cymbulifera* prefer to grow in homogeneous habitats that are often humid, and *L. tongolensis* inhabit diverse habitats (X. Gong, personal observation). The putative hybrids between the putative parents were commonly found near Zhongdian, where the two species share a sympatric existence. The wintry highland climate requires species to evolve certain common adaptive mechanisms. *Ligularia* species often share the same pollinators in the highlands [32], [33]. *L. vellerea*, *L. tongolensis* and *L. cymbulifera* flower from July to August [9], [19]. According to our field observations, nectar-collecting insects had no species preference within *Ligularia* and shuttled frequently among flowers. This behavior was highly likely to transfer one species' pollen to another species' flowers. Because the three species have nearly the same peak flowering times, it is difficult to reject any of them as possible participants in interspecific hybridization.

Based on data of ITS4-5 and three cpDNA, *L. vellerea* was absolutely uninvolved in natural hybridization in these mixed populations. The ITS network definitely supports the hypothesis that the morphologically intermediate individuals are the products of hybridization between *L. tongolensis* and *L. cymbulifera*.

### Diversity of natural hybridization

Because chloroplast DNA is maternally inherited in *Ligularia* [29], we expected that the cpDNA sequences could determine the direction of natural hybridization between *L. tongolensis* and *L. cymbulifera*. Yu et al. [15] studied natural hybridization between *Ligularia subspicata* and *L. nelumbifolia* in a mixed location where the directions support the prediction that the minority species usually acts as the maternal parent. Zhou et al. [34] argued for the opposite opinion based on three different hybridizing taxa. Here, we anticipated that the four different mixed locations would verify the hybrid direction.

In Pachahai and Jiawa, the numbers of *Ligularia cymbulifera* and *L. tongolensis* were almost the same (*L. vellerea* were not involved in hybridization, although they were sympatric). Morphological classifications were mostly in accordance with molecular results except for three individuals (Pachahai) and two individuals (Jiawa) of *L. tongolensis*, which proved to be introgressed individuals. In Pachahai, four hybrids shared a cpDNA haplotype (H12) with pure *L. cymbulifera*, and six other hybrids shared a common haplotype (H7) with both pure parents. In Jiawa, half of the ten hybrids shared a cpDNA haplotype (H4) with both pure parents, and another four hybrids showed three particular haplotypes that were not detected elsewhere. It is certain only that *L. cymbulifera* acted as a chloroplast donor (Pachahai). The complementary direction may exist but was not detected because of hybridization and introgression.

In Desha, the exceptional case in our field research, it was extremely difficult to identify typical *L. cymbulifera*, and almost all the collections were *L. tongolensis* and putative hybrids. Only the three *L. cymbulifera*-like collections actually proved to be introgressed products. One hybrid and two *L. cymbulifera*-like collections shared a cpDNA haplotype (H2) with most of the reference *L. cymbulifera* in Suochonghe, which was not too far from Desha; nine hybrids shared a haplotype (H4) with one pure *L. tongolensis*. We can infer that hybridization was initially bidirectional but occurred predominantly in the form of *L. tongolensis* ♀ × *L. cymbulifera* ♂. The hybridization and introgression may continue for a long time, so the actual process cannot be determined with existing methods.

In Xiaoxueshan, although *L. cymbulifera* and *L. tongolensis* were almost equally represented (*L. vellerea* were not involved in hybridization, although sympatric), the site was previously dominated by fir and was destroyed by a fire during the 1980s. In the network, this near equality was unique to Xiaoxueshan. Except for three individuals of *L. cymbulifera* that shared the most common haplotype (H7), other collections at this site revealed population-specific haplotypes. Most *L. tongolensis* shared haplotype H21, and most hybrids shared haplotype H18, which was the closest to H21 in the network. Most likely, the chloroplast donor at the initial instance of hybridization was *L. tongolensis*.

In summary, natural hybridization and also introgression between sympatric *L. cymbulifera* and *L. tongolensis* occurred in multiple places and diverse forms. However, the direction of hybridization is not simply correlated with the number of parent individuals. Many factors, such as differences in flowering times, floral organs, pollen tube germination on different stigmas, and the ratio of mature individuals, may codetermine the direction of hybridization.

### Effects on putative parents

Continuous hybridization may affect the independence of the parental taxa, even resulting in reticulate evolution. For the parental species *L. cymbulifera*, morphological identifications were generally in accordance with the molecular results, except for Desha, which had introgressed individuals. For *L. tongolensis*, except in Desha, another three mixed locations had a portion of introgressed individuals. Based on ITS data, all the morphological and pure *L. cymbulifera* had low ITS sequence biodiversity, and *L. tongolensis* had relatively high ITS sequence variability. The results were consistent with the results of a study of chemical and *atp*B-*rbc*L data for 19 individuals of *L. tongolensis* and 13 individuals of *L. cymbulifera* from different plots [35].

With respect to the three cpDNA sequences, the network was disordered and unintelligible. Part of our previous study on the barcode *Ligularia* indicated that *L. cymbulifera* and *L. tongolensis* had identical sequences for three optional sequences (*mat*K, *rbc*L, *psb*A-*trn*H) (GenBank accession numbers: JF954335-JF954341, JF954362-JF954365, JF942246-JF942252, JF942273-JF942276, JN045182-JN045188, JN045209-JN045212). Therefore, three other fragments were selected in the present study. The *trn*K-*rps*16, *trn*L-*rpl*32 and *trn*Q*-*5'*rp*s16 spacers were known to be more variable than the other chloroplast regions [23]. This also occurred in *L. cymbulifera* and *L. tongolensis*. However, the three spacers still generated a problematic network. There are several reasons for the difficulty in clarifying the phylogeny: (A) convergent mutations may have caused the same haplotype to arise independently in different species; (B) shared haplotypes may have been inherited from a polymorphic common ancestor due to incomplete lineage sorting; and (C) one species may share haplotypes with another species via introgressive hybridization within a framework of stable species ranges, perhaps assisted by selection. The genus *Ligularia* has been estimated to have occurred primarily within the last 20 million years, and interspecific diploid hybridization has played a role in the species diversity of this genus [11]. The three spacers we selected may vary significantly in a relatively short evolutionary time period and could not give the expected reliable phylogeny. As is known, convergence is intrinsically unlikely at neutral loci, and if it did occur, it would be expected to yield symmetric allele sharing between the putative parents *L. cymbulifera* and *L. tongolensis*. However, based on a study by Hanai et al. [35], no haplotypes are shared between *L. cymbulifera* and *L. tongolensis* in different populations. Our ITS data in allopatric reference populations also confirmed this pattern. Accordingly, the explanations of convergent mutations (A) and incomplete lineage sorting (B) can be excluded in the present study. In addition, our ITS data do show that natural hybridization and introgression occurred between sympatric *L. cymbulifera* and *L. tongolensis*.

Species distinctiveness is maintained if hybrids are selected by extrinsic and intrinsic selection despite continuous gene flow [36]. In the present study, the putative parents remained distinct from each other, but the genetic diversity increased, e.g., *L. tongolensis* in Pachahai and Jiawa. The diverse hybrid individuals may adapt to more niches and form new taxa, as is the case for hybridization in sunflowers [37]. The data show that hybridization does occur in *Ligularia*, but more credible measures, such as SSR, spot investigation, and artificial hybrid experiments, would help provide a better understanding of the way in which hybridization occurred and affected species evolution.

## Conclusions

Hybridization is very frequent in *Ligularia*, appearing between different species, series and subgenera. In this case, the direct and cloned ITS sequences and the three chloroplast regions of the individuals in four populations confirmed that hybridization occurs between *L. cymbulifera* and *L. tongolensis*, and this phenomenon was demonstrated simultaneously in several locations separated by long distances. Although gene flow is common in *Ligularia*, most species maintain their independence. Because of the lack of study of reproductive isolation in *Ligularia* and selection on hybrid descendants, the impact of natural hybridization on the evolution and speciation of *Ligularia* is still not clear. Future work should focus on comparing the fitness of hybrids and their parental species and on considering the effect of natural hybridization.

## Supporting Information

S1 File **Table S1, Sample locations for 4 sympatric locations and 2 reference** ***Ligularia*** **populations.** Table S2, Variable sites from the aligned sequences (both direct sequencing and cloned sequencing) of ITS4-5 in the 15 haplotypes (H1-H15) of all the collections. Table S3, Variable sites from the aligned sequences of the three chloroplast DNA spaces in the 22 haplotypes (H1-H22) of all the collections.
(DOCX)

S2 File **Figure of sample locations for the four mixed populations (dotted area) and two reference populations (triangular area) of** ***Ligularia*** **from south-western China.**
(DOCX)
