# Supplementary material for: Natural Hybridization and Introgression between Ligularia cymbulifera and L. tongolensis (Asteraceae, Senecioneae) in Four Different Locations
Source: PLoS One. 2014 Dec 31;9(12):e115167. doi: 10.1371/journal.pone.0115167 (PMC4281107; doi:10.1371/journal.pone.0115167)
Supplement: S1 File — Table S1, Sample locations for 4 sympatric locations and 2 reference Ligularia populations. Table S2, Variable sites from the aligned sequences (both direct sequencing and cloned sequencing) of ITS4-5 in the 15 haplotypes (H1-H15) of all the collections. Table S3, Variable sites from the aligned sequences of the three chloroplast DNA spaces in the 22 haplotypes (H1-H22) of all the collections. (DOCX) [file pone.0115167.s001.docx]

**
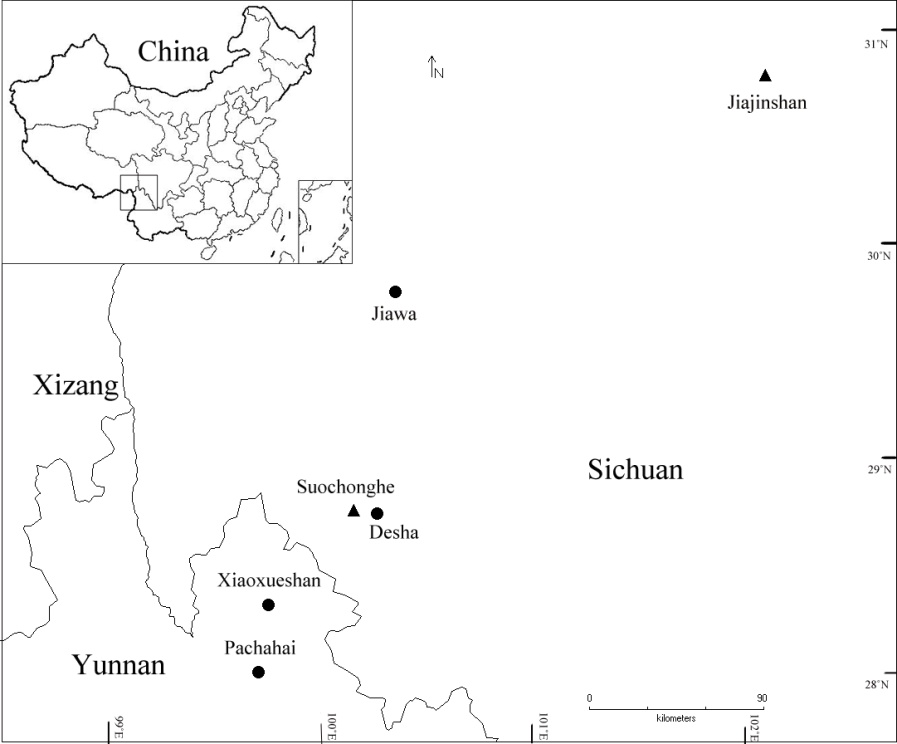
**

Figure S1. Sample locations for the four mixed locations (dotted area) and two reference populations (triangular area) of *Ligularia* from southwestern China.
