# Supplementary material for: Natural Hybridization and Introgression between Ligularia cymbulifera and L. tongolensis (Asteraceae, Senecioneae) in Four Different Locations
Source: PLoS One. 2014 Dec 31;9(12):e115167. doi: 10.1371/journal.pone.0115167 (PMC4281107; doi:10.1371/journal.pone.0115167)
Supplement: S2 File — Figure of sample locations for the four mixed populations (dotted area) and two reference populations (triangular area) of Ligularia from south-western China. (DOCX) [file pone.0115167.s002.docx]

Table S1 Sample locations for 4 sympatric locations and 2 reference *Ligularia* populations

| Locality | Latitude（N） | Longitude（E） | Altitude（m） | Taxa | Vouchers | Sampling | Labels |
| --- | --- | --- | --- | --- | --- | --- | --- |
| 1.Jiajinshan, Baoxing (Sichuan) | 30˚49΄34.5˝ | 102˚42΄42.8˝ | 3300 | *Ligularia tongolensis* | PG110803 | 12 | RJt1, RJt2, RJt3, RJt4R, Jt5, RJt6, RJt7, RJt8, RJt9, RJt10 |
| 2.Suochonghe, Daocheng (Sichuan) | 28˚44΄46.8˝ | 100˚16΄08.7˝ | 3700 | *L.cymbulifera* | PG110859 | 24 | RDc1, RDc2, RDc3, RDc4, RDc5, RDc6, RDc7, RDc8, RDc9, RDc10 |
| 3.Desha, Daocheng (Sichuan) | 28˚44΄46.8˝ | 100˚16΄08.7˝ | 3900 | *L. tongolensis* | PG110856 | 22 | Dt1, Dt2, Dt3, Dt4, Dt5, Dt6, Dt7, Dt8, Dt9, Dt10 |
|  |  |  |  | *L. cymbulifera* | PG110855 | 3^*^ | Dc1, Dc2, Dc3 |
|  |  |  |  | putative hybrids | PG110857 | 15 | Dh1, Dh2, Dh3, Dh4, Dh5, Dh6, Dh7, Dh8, Dh9, Dh10, |
| 4.Pachahai, Zhongdian (Yunnan) | 27˚59΄39.2˝ | 99˚42΄14.5˝ | 3500 | *L. tongolensis* |  | 30 | Pt1, Pt2, Pt3, Pt4, Pt5, Pt6, Pt7, Pt8, Pt9, Pt10 |
|  |  |  |  | *L. cymbulifera* |  | 13 | Pc1, Pc2, Pc3, Pc4, Pc5, Pc6, Pc7, Pc8, Pc9, Pc10 |
|  |  |  |  | putative hybrids | PG110866 | 26 | Ph1, Ph2, Ph3, Ph4, Ph5, Ph6, Ph7, Ph8, Ph9, Ph10 |
|  |  |  |  | *L. vellerea* |  | 15 | Pv1, Pv2, Pv3, Pv4, Pv5, Pv6, Pv7, Pv8, Pv9, Pv10 |
| 5.Xiaoxueshan, Zhongdian (Yunnan) | 28˚18΄53.4˝ | 99˚45΄11.4˝ | 3800 | *L. tongolensis* |  | 12 | Xt1, Xt2, Xt3, Xt4, Xt5, Xt6, Xt7, Xt8, Xt9, Xt10 |
|  |  |  |  | *L. cymbulifera* |  | 10 | Xc1, Xc2, Xc3, Xc4, Xc5, Xc6, Xc7, Xc8, Xc9, Xc10 |
|  |  |  |  | putative hybrids |  | 24 | Xh1, Xh2, Xh3, Xh4, Xh5, Xh6, Xh7, Xh8, Xh9, Xh10 |
|  |  |  |  | *L. vellerea* |  | 4* | Xv1, Xv2, Xv3, Xv4, Xv5 |
| 6.Jiawa, Litang (Sichuan) | 29˚47΄51.2˝ | 100˚21΄21.5˝ | 3800 | *L. tongolensis* | PG090949 | 25 | Lt1, Lt2, Lt3, Lt4, Lt5, Lt6, Lt7, Lt8, Lt9, Lt10 |
|  |  |  |  | *L. cymbulifera* | PG090950 | 27 | Lc1, Lc2, Lc3, Lc4, Lc5, Lc6, Lc7, Lc8, Lc9, Lc10 |
|  |  |  |  | putative hybrids | PG090951 | 35 | Lh1, Lh2, Lh3, Lh4, Lh5, Lh6, Lh7, Lh8, Lh9, Lh10 |

Note:*indicates that only three or four individuals for this taxon were found within the population.

Table S2 Variable sites from the aligned sequences (both direct sequencing and cloned sequencing) of ITS4-5 in the 15 haplotypes (H1-H15) of all collections

|  | ITS4-5 Polymorphic sites | | | | | | | | | | | | | | | | | | | | | | | |
| --- | --- | --- | --- | --- | --- | --- | --- | --- | --- | --- | --- | --- | --- | --- | --- | --- | --- | --- | --- | --- | --- | --- | --- | --- |
| Haplotype | 3  8 | 65  -  66 | 7    5 | 9  6 | 1  0  0 | 1  1  6 | 1  4  6 | 1  5  3 | 1  5  5 | 1  6  6 | 1  7  0 | 1  7  4 | 179  -  181 | 1  8  3 | 1  8  6 | 1  9  8 | 2  0  2 | 2  0  8 | 2  2  1 | 2  3  2 | 2  5  0 | 2  6  3 | 2  6  9 | 2  7  5 |
| H1 | C | CT | C | T | C | C | G | T | C | A | C | G | CTC | G | T | A | C | C | A | G | A | T | C | G |
| H2 | C | CT | C | T | C | C | G | C | A | A | C | G | CTC | G | T | A | C | C | A | G | A | T | C | G |
| H3 | G | CT | C | T | C | C | G | T | C | A | C | G | CTC | G | T | A | C | C | A | G | A | T | C | G |
| H4 | C | CT | C | T | C | C | G | T | C | A | C | G | GTC | G | T | A | C | C | A | G | A | T | C | G |
| H5 | C | CT | C | T | C | C | T | T | C | A | C | G | CTC | C | T | A | C | C | A | G | A | T | C | G |
| H6 | C | CT | C | T | C | T | G | T | C | A | C | G | CTC | G | T | A | C | C | A | G | A | T | C | G |
| H7 | C | CT | C | T | C | C | G | T | C | A | C | G | CTC | G | T | A | C | C | A | G | A | T | C | G |
| H8 | C | TC | T | T | C | C | G | T | C | G | T | G | CTA | G | T | T | C | C | C | T | A | T | T | G |
| H9 | C | CC | C | T | C | C | G | T | C | G | T | G | CTA | G | C | T | C | C | C | G | A | T | T | G |
| H10 | C | CC | C | T | C | C | G | T | C | G | T | G | CTA | G | C | T | C | C | C | G | A | T | T | G |
| H11 | C | CC | C | T | C | C | G | T | C | G | T | G | CTA | G | C | T | C | C | C | G | A | T | T | G |
| H12 | C | CC | C | T | C | C | G | T | C | G | T | G | CTA | G | C | T | C | C | C | G | A | T | T | G |
| H13 | C | CT | C | T | C | C | G | T | C | G | T | G | CTA | G | C | T | C | C | C | G | A | T | T | G |
| H14 | C | CC | C | T | C | C | G | T | C | G | T | G | CTA | G | C | T | C | C | C | G | A | T | T | G |
| H15 | C | CA | C | C | T | C | G | T | C | A | C | A | CTA | G | C | G | T | A | C | G | G | G | C | A |

Note: C+T=Y, A+C=M, A+T=W, A+G=R, G+T=K, C+G=S.

Continuation of Table S2. Variable sites from the aligned sequences (both direct sequencing and cloned sequencing) of ITS4-5 in the 15 haplotypes (H1-H15) of all collections

|  | ITS4-5 | | | | | | | | | | | | | | | | | | | | | | | | |
| --- | --- | --- | --- | --- | --- | --- | --- | --- | --- | --- | --- | --- | --- | --- | --- | --- | --- | --- | --- | --- | --- | --- | --- | --- | --- |
| Haplotype | 2 8 5 | 291  -  293 | 2  9  5 | 4  3  4 | 4 4 3 | 4 4 7 | 4 7 7 | 4 8 8 | 4  8  9 | 4 9 3 | 5 0 1 | 5  0  6 | 5  1  0 | 5  1  9 | 5  4  2 | 5 7 9 | 5 8 1 | 5 8 5 | 5 8 9 | 6  0  6 | 6  3  0 | 6  4  8 | 6 7 3 | 6 8 3 | 694  -  695 |
| H1 | C | TCG | G | G | T | T | C | C | A | C | C | G | T | A | A | C | C | C | C | A | A | T | T | C | CT |
| H2 | C | TCG | G | G | T | T | C | C | A | C | C | G | T | A | A | C | C | C | C | A | A | T | T | C | CT |
| H3 | C | TCG | G | G | T | T | C | C | A | C | C | G | T | A | A | C | C | C | C | A | A | T | T | C | CT |
| H4 | C | TCG | G | G | T | T | C | C | A | C | C | G | T | A | A | C | C | C | C | A | A | T | T | C | CT |
| H5 | C | TCA | G | G | T | T | C | C | A | C | C | G | T | A | A | C | C | C | C | A | A | T | T | C | CT |
| H6 | C | TCA | G | G | T | T | C | C | A | C | C | G | T | A | A | C | C | C | C | A | A | T | T | C | CT |
| H7 | C | ACA | G | G | T | T | C | C | A | C | C | G | T | A | A | C | C | C | C | A | A | T | T | C | CT |
| H8 | T | ACA | G | A | T | T | C | C | G | T | C | G | A | G | A | C | T | C | C | A | A | G | T | T | TT |
| H9 | T | ACA | G | G | T | T | T | C | A | T | C | G | A | G | A | C | C | C | T | A | A | G | T | T | TT |
| H10 | T | ACA | G | G | T | T | T | C | A | T | C | G | A | G | A | C | C | C | T | A | A | G | T | T | TT |
| H11 | T | ACA | G | G | T | T | T | C | A | T | C | G | A | G | A | C | C | C | C | A | A | G | T | T | TT |
| H12 | T | ACA | G | G | T | T | T | C | A | T | T | G | A | G | A | C | C | C | C | A | A | G | T | T | TT |
| H13 | T | ACA | G | G | T | T | T | C | A | T | T | G | A | G | A | C | C | C | C | A | A | G | T | T | TT |
| H14 | T | ACA | G | G | T | T | C | C | A | T | C | G | A | G | A | C | C | C | C | A | A | G | T | C | CT |
| H15 | C | ACG | A | G | C | C | C | C | A | C | C | A | T | G | T | T | C | T | C | C | G | T | C | C | CC |

Note: C+T=Y, A+C=M, A+T=W, A+G=R, G+T=K, C+G=S.

Table S3 Variable sites from the aligned sequences of the three chloroplast DNA spaces in the 27 haplotypes of all collections

| Haplotype |  | *trn*K-*rps*16 | | | | | | |  | *trn*L-*rpl*32 | | | | | | | | | | |  | *trn*Q-5'*rps*16 | | | | | | | | | | | |
| --- | --- | --- | --- | --- | --- | --- | --- | --- | --- | --- | --- | --- | --- | --- | --- | --- | --- | --- | --- | --- | --- | --- | --- | --- | --- | --- | --- | --- | --- | --- | --- | --- | --- |
|  | 2 4 9 | 405 - 410 | 7 2 8 | 755 - 791 | 7  9 5 | 8 0 9 | 8  1 5 | 8 5 4 |  | 9  5 | 1  5 0 | 1  6 2 | 355  -  356 | 4  1 9 | 4  2 4 | 484  -  486 | 533  -  537 | 6  0 7 | 6 7 0 | 8 3 7 |  | 1  4 1 | 1  8 5 | 266  -  268 | 2  7 6 | 2  9 6 | 407  -  408 | 4  6 0 | 5  1 5 | 5 2 6 | 5  3 5 | 6  3 1 | 8  5 5 |
| H1 | G | — | **—** | ② | G | — | C | — |  | — | T | C | T- | C | G | — | — | G | — | T |  | G | C | — | A | A | TT | A | A | A | A | C | T |
| H2 | G | **—** | **—** | ② | G | — | C | — |  | — | T | C | T- | C | G | — | — | G | — | T |  | G | C | — | A | A | TT | A | A | — | A | C | T |
| H3 | G | **—** | **—** | ② | G | — | C | — |  | — | T | A | T- | C | G | — | — | G | A | T |  | G | C | — | A | A | AG | A | C | A | A | C | T |
| H4 | G | **—** | **—** | ② | G | — | C | — |  | — | T | A | T- | C | G | — | — | G | — | T |  | G | C | — | A | A | TT | A | A | — | A | C | T |
| H5 | G | **—** | **—** | ② | G | — | C | — |  | — | T | A | T- | C | G | — | — | G | A | T |  | A | A | — | A | A | AG | A | C | A | A | T | A |
| H6 | G | **—** | **—** | ② | G | — | C | — |  | — | T | A | T- | C | G | — | — | T | A | T |  | G | C | — | A | A | AG | A | C | A | A | C | T |
| H7 | G | **—** | **—** | ② | G | — | C | — |  | — | T | A | T- | C | G | — | — | G | — | T |  | G | C | — | A | A | TT | A | A | A | A | C | T |
| H8 | G | **—** | **—** | ② | G | — | C | — |  | — | T | A | T- | C | G | — | — | G | — | T |  | G | C | — | A | A | AG | A | C | A | A | C | T |
| H9 | G | **—** | **—** | ② | G | — | C | — |  | — | T | A | T- | C | G | — | — | G | — | — |  | G | C | — | A | A | TT | A | A | — | A | C | T |
| H10 | G | **—** | **—** | ② | G | — | C | — |  | — | T | A | T- | C | G | — | — | G | A | — |  | G | C | — | A | A | AG | A | C | A | A | C | T |
| H11 | G | **—** | **—** | ② | G | — | C | — |  | — | T | A | T- | C | G | — | — | G | A | — |  | G | C | — | A | A | TT | A | A | — | A | C | T |
| H12 | G | **—** | **—** | ② | G | — | C | — |  | — | T | A | T- | C | G | — | — | G | — | T |  | G | C | — | A | A | TT | A | A | A | A | C | T |
| H13 | G | **—** | **—** | ② | G | — | T | — |  | — | T | A | T- | C | G | — | — | G | A | T |  | G | C | — | A | A | TT | A | C | A | T | C | T |
| H14 | G | **—** | **—** | ② | G | — | C | — |  | — | T | A | T- | C | G | — | — | G | — | T |  | G | C | — | C | A | TT | A | A | A | A | C | T |

Note: —, deletions; +, insertions;

①: CAATA (5bp); ②: TTATTTATTTGATCAGTAAATTTTTTATTTTTT-CAT; ③: TTATTTATTTGATCAGTAAATTTTTTATTTTTTCAT (36bp); ④: CAT (3bp); ⑤: ATATT (5bp); ⑥:TAA (3bp).

Continuation of Table S3. Variable sites from the aligned sequences of the three chloroplast DNA spaces in the 27 haplotypes of all collections

| Haplotype |  | *trn*K-*rps*16 | | | | | | |  | *trn*L-*rpl*32 | | | | | | | | | | |  | *trn*Q-5'*rps*16 | | | | | | | | | | | |
| --- | --- | --- | --- | --- | --- | --- | --- | --- | --- | --- | --- | --- | --- | --- | --- | --- | --- | --- | --- | --- | --- | --- | --- | --- | --- | --- | --- | --- | --- | --- | --- | --- | --- |
|  | 2 4 9 | 405 - 410 | 7 2 8 | 755 - 791 | 7 9 5 | 8 0 9 | 8 1 5 | 8 5 4 |  | 9  5 | 1 5 0 | 1 6 2 | 355  -  356 | 4 1 9 | 4 2 4 | 484  -  486 | 533  -  537 | 6 0 7 | 6 7 0 | 8 3 7 |  | 1 4 1 | 1 8 5 | 266  -  268 | 2 7 6 | 2 9 6 | 407  -  408 | 4 6 0 | 5 1 5 | 5 2 6 | 5 3 5 | 6 3 1 | 8 5 5 |
| H15 | G | ① | **—** | — | G | — | C | — |  | — | T | A | T- | C | G | — | — | G | — | T |  | G | C | — | C | A | TT | A | A | A | A | C | T |
| H16 | G | **—** | **—** | ③ | G | — | C | T |  | — | T | A | T- | C | G | — | — | G | A | T |  | G | C | — | C | A | TT | A | A | A | A | C | T |
| H17 | G | **—** | **—** | ③ | G | — | C | T |  | — | T | A | T- | C | G | — | — | G | — | T |  | G | C | — | C | A | TT | A | A | A | A | C | T |
| H18 | G | ① | **—** | — | G | — | C | — |  | — | G | A | AA | C | G | — | ⑤ | G | — | T |  | G | C | ⑥ | A | G | TT | — | C | — | A | C | T |
| H19 | G | ① | **—** | — | G | T | C | — |  | C | G | A | AA | C | G | — | ⑤ | G | — | T |  | G | C | ⑥ | A | G | TT | — | C | — | A | C | T |
| H20 | G | ① | **—** | — | G | — | C | — |  | C | T | A | T- | C | G | — | — | G | A | T |  | G | C | — | A | A | TT | A | C | A | T | C | T |
| H21 | G | ① | **—** | — | G | — | C | — |  | — | G | A | AA | C | G | — | ⑤ | G | — | T |  | G | C | ⑥ | A | G | TT | — | C | — | A | C | T |
| H22 | G | ① | **—** | — | G | T | C | — |  | — | G | A | AA | C | G | — | ⑤ | G | — | T |  | G | C | ⑥ | A | G | TT | — | C | — | A | C | T |
| H23 | G | **—** | A | ② | G | — | C | — |  | — | T | A | T- | C | G | ④ | — | G | A | T |  | G | C | — | A | A | AG | A | C | — | A | C | T |
| H24 | G | **—** | **—** | ② | G | — | C | — |  | — | T | A | T- | C | G | ④ | — | G | A | T |  | G | C | — | A | A | AG | A | C | — | A | C | T |
| H25 | A | **—** | **—** | ② | G | — | C | — |  | — | T | A | T- | C | G | ④ | — | G | A | T |  | G | C | — | A | A | AG | A | C | — | A | C | T |
| H26 | G | **—** | **—** | ② | G | — | C | — |  | — | T | A | T- | A | A | ④ | — | G | A | T |  | G | C | — | A | A | AG | A | C | — | A | C | T |
| H27 | A | **—** | **—** | ② | A | — | C | — |  | — | T | A | T- | C | G | ④ | — | G | A | T |  | G | C | — | A | A | AG | A | C | — | A | C | T |

Note: —, deletions; +, insertions;

①: CAATA (5bp); ②: TTATTTATTTGATCAGTAAATTTTTTATTTTTT-CAT; ③: TTATTTATTTGATCAGTAAATTTTTTATTTTTTCAT (36bp); ④: CAT (3bp); ⑤: ATATT (5bp); ⑥:TAA (3bp).
